# Supplementary figures and images for: Antibody responses to Zika virus proteins in pregnant and non-pregnant macaques
Source: PLoS Negl Trop Dis. 2018 Nov 27;12(11):e0006903. doi: 10.1371/journal.pntd.0006903 (PMC6286021; doi:10.1371/journal.pntd.0006903)

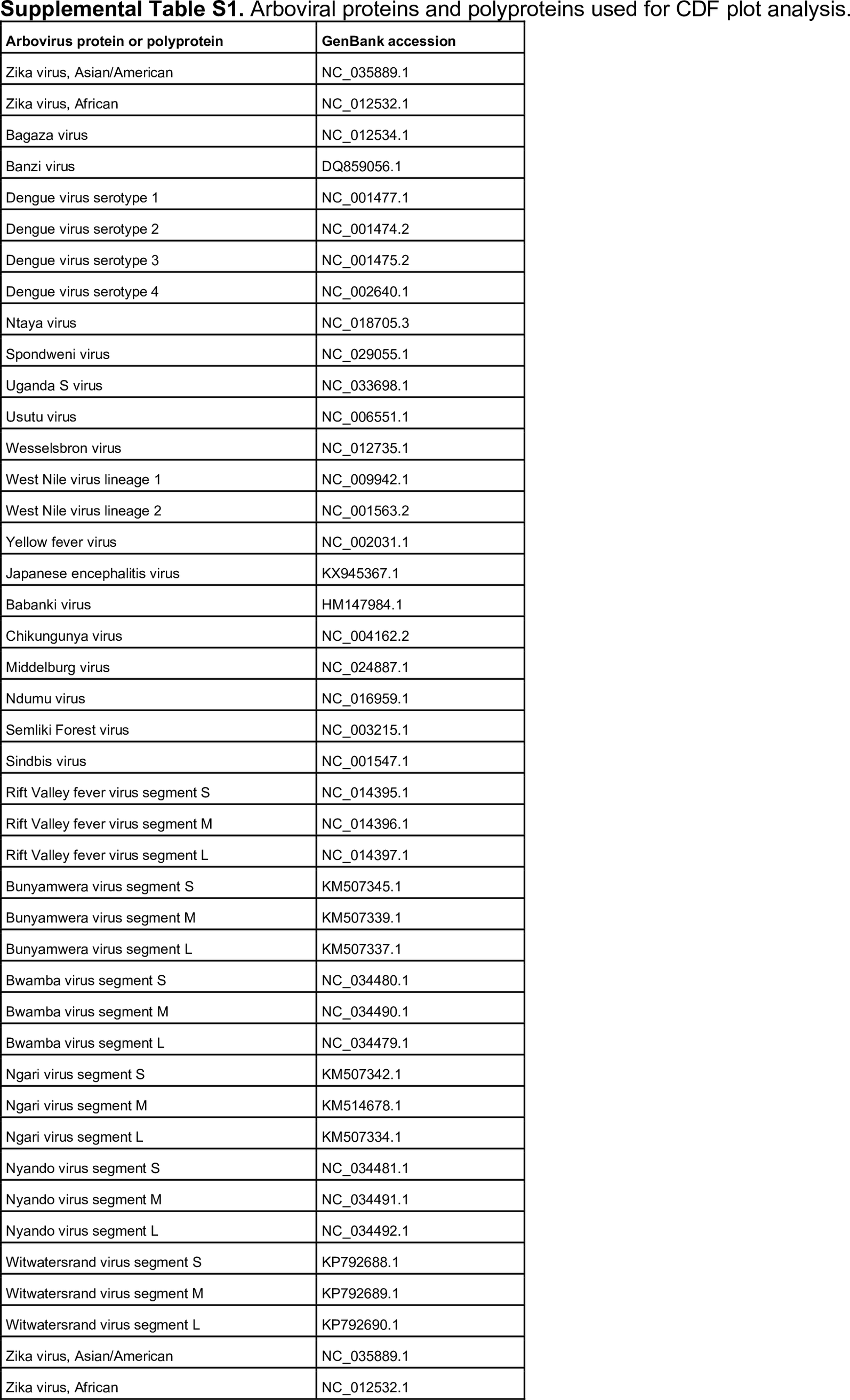

Supplement: S1 Table — (TIF) [file pntd.0006903.s001.tif]

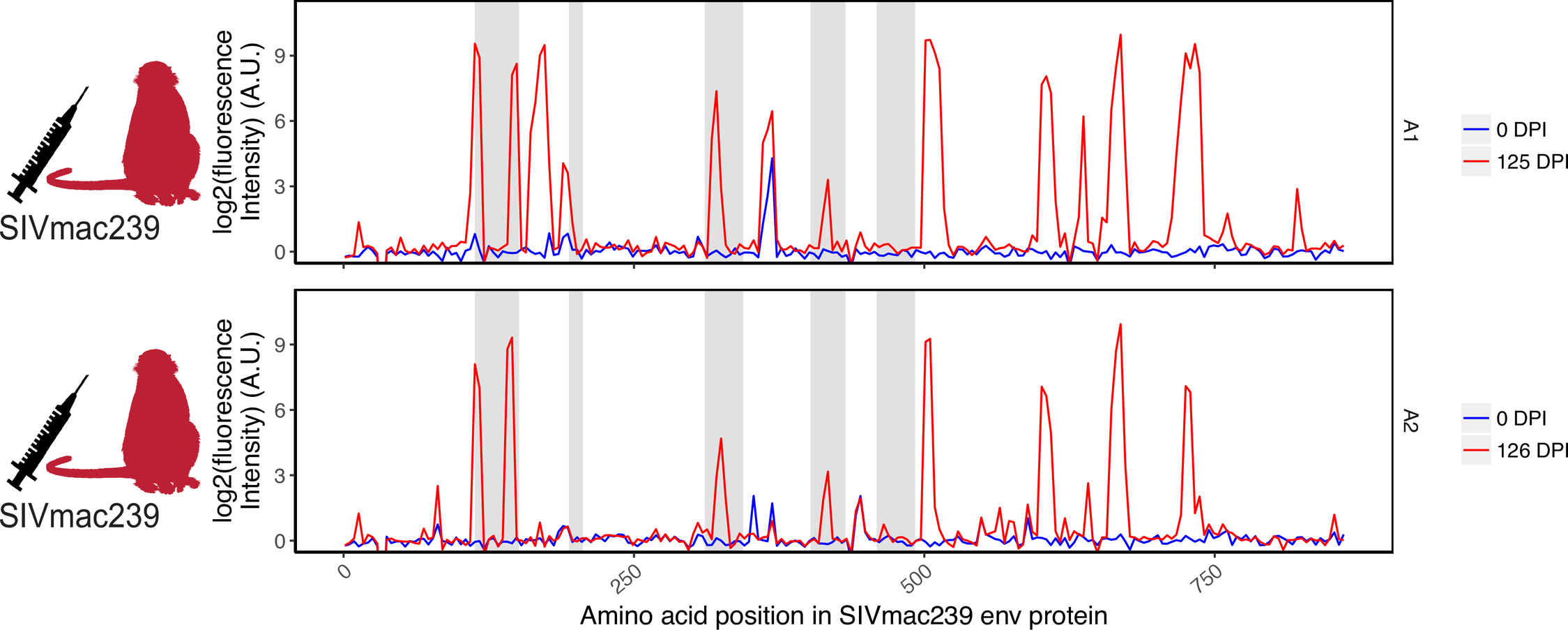

Supplement: S1 Fig — Serum from two Mauritian cynomolgus macaques (animals A1 and A2) before and after infection with SIVmac239 was evaluated for reactivity to overlapping peptides representing the SIVmac239 env protein sequence on a linear peptide microarray. SIV env variable loop regions [41] are highlighted in grey. (TIF) [file pntd.0006903.s002.tif]

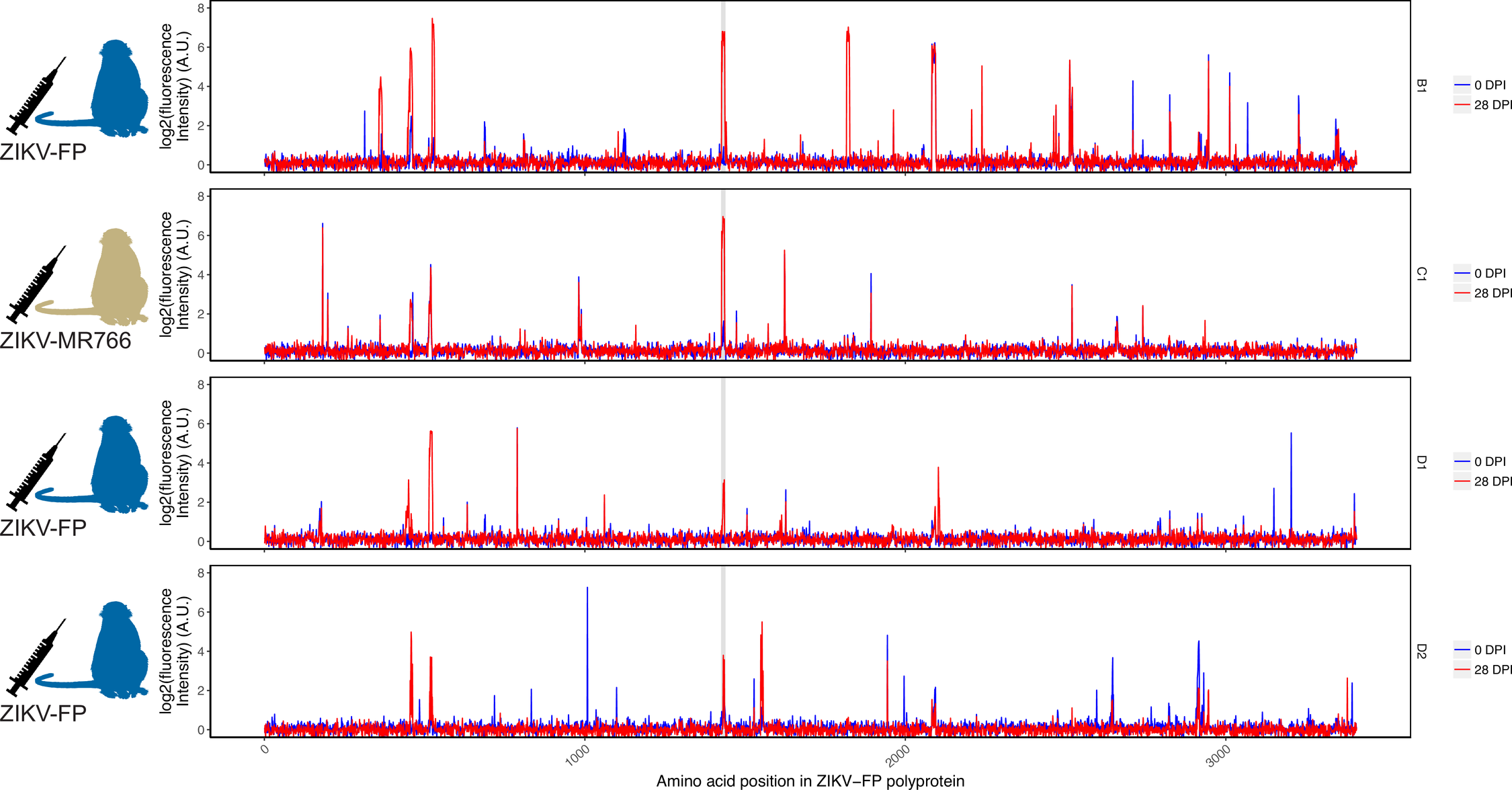

Supplement: S2 Fig — The NS2B1427-1451RD25 epitope is highlighted in grey. (TIF) [file pntd.0006903.s003.tif]

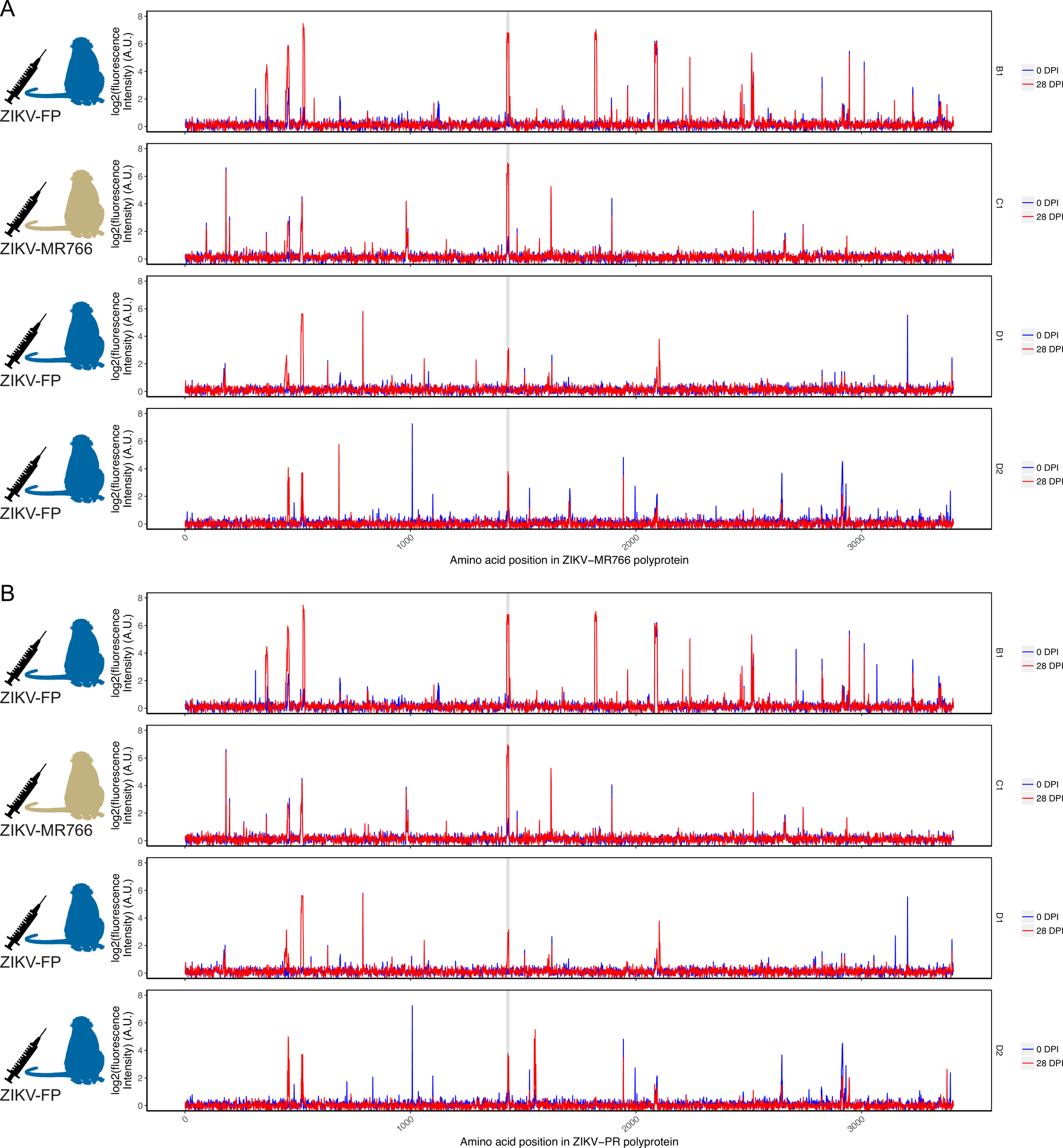

Supplement: S3 Fig — The NS2B1427-1451RD25 epitope is highlighted in grey. (TIF) [file pntd.0006903.s004.tif]

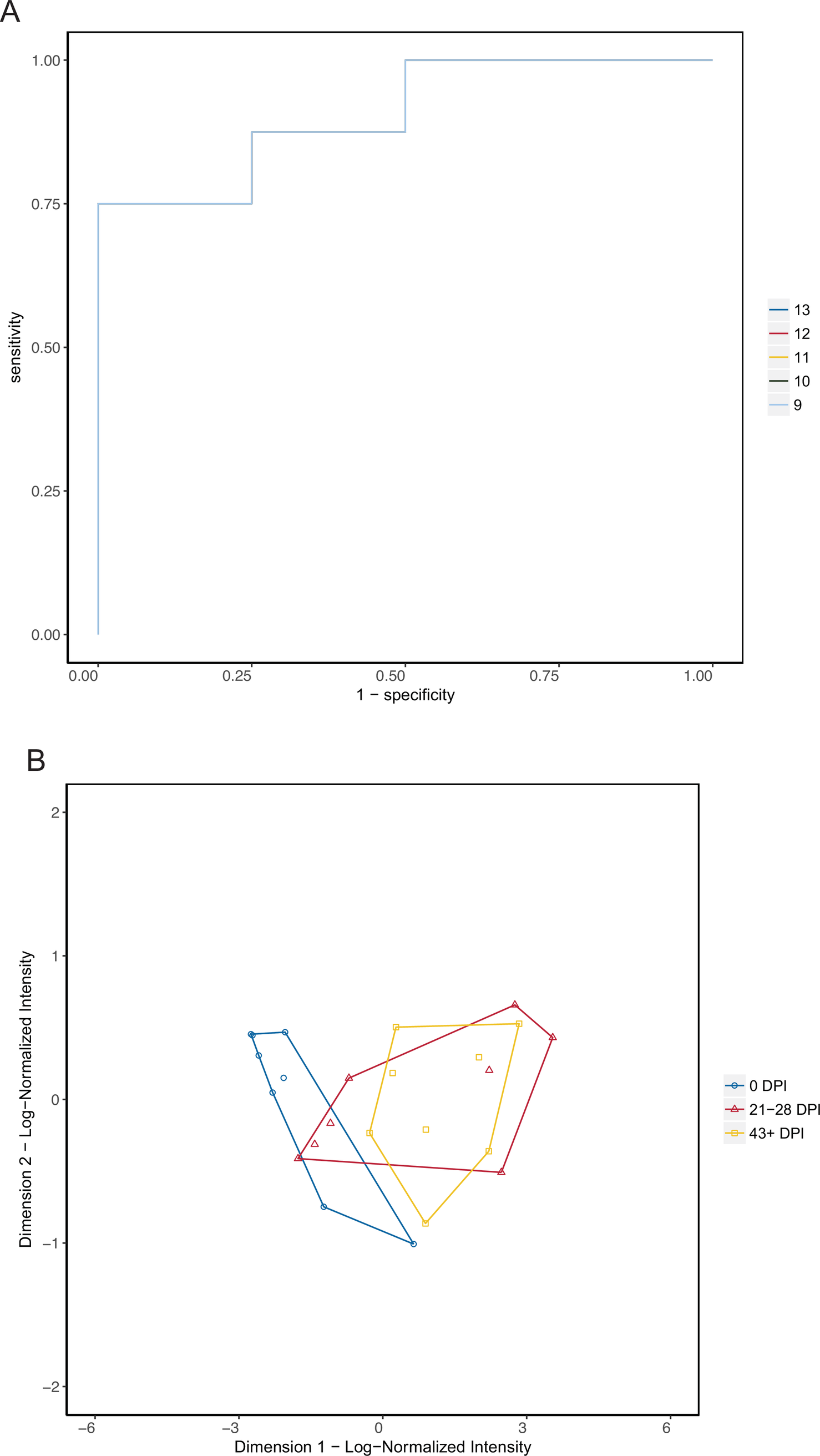

Supplement: S4 Fig — ROC curves (A) were generated using data from the 8 animals analyzed against ZIKV polyproteins tiled as 16 amino acid peptides overlapping by 15 amino acids since this was the most complete dataset. ROC curves were created using the 8 samples collected at 0 DPI as the control group and the same 8 samples collected at 21–28 DPI samples as the test group. We chose consecutive peptides within the NS2B region that maximized the differences of mean log-normalized intensities between the controls and test samples. The decision threshold was determined by maximizing the AUC and the resulting ROCs of 9 to 13 peptides were plotted. ROC curves overlapped. MDS plots (B) of distances between the log-normalized gene expression profiles were created using Limma plot MDS R library. Only the 10 peptides in the identified NS2B1427-1451RD25 epitope were used. Based on MDS analysis, pre-infection samples (blue), samples from early convalescence (21–28 dpi, red), and samples from any later time (>43 dpi, yellow) clustered in separate groups. (TIF) [file pntd.0006903.s005.tif]

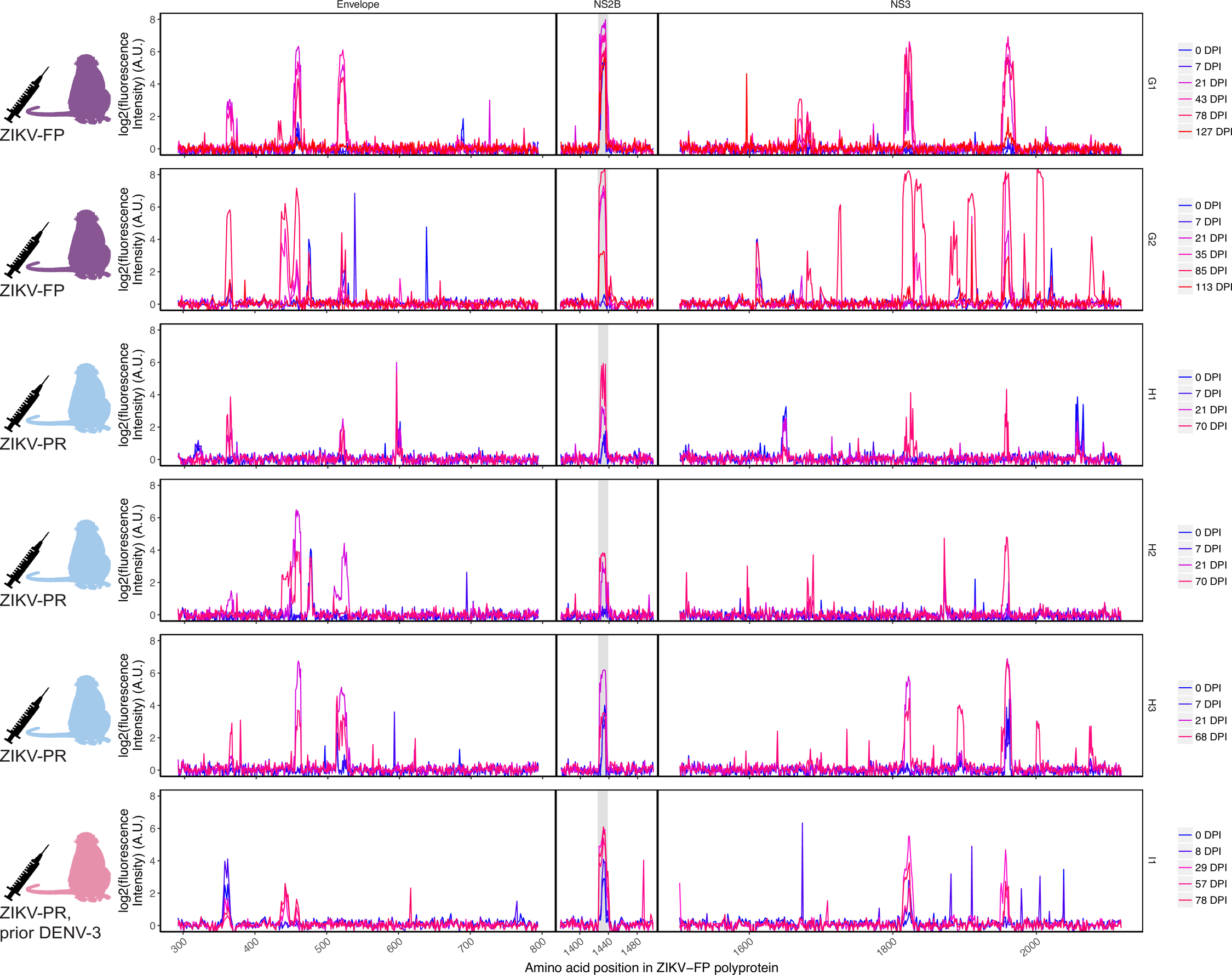

Supplement: S5 Fig — The NS2B1427-1451RD25 epitope is highlighted in grey. (TIF) [file pntd.0006903.s006.tif]

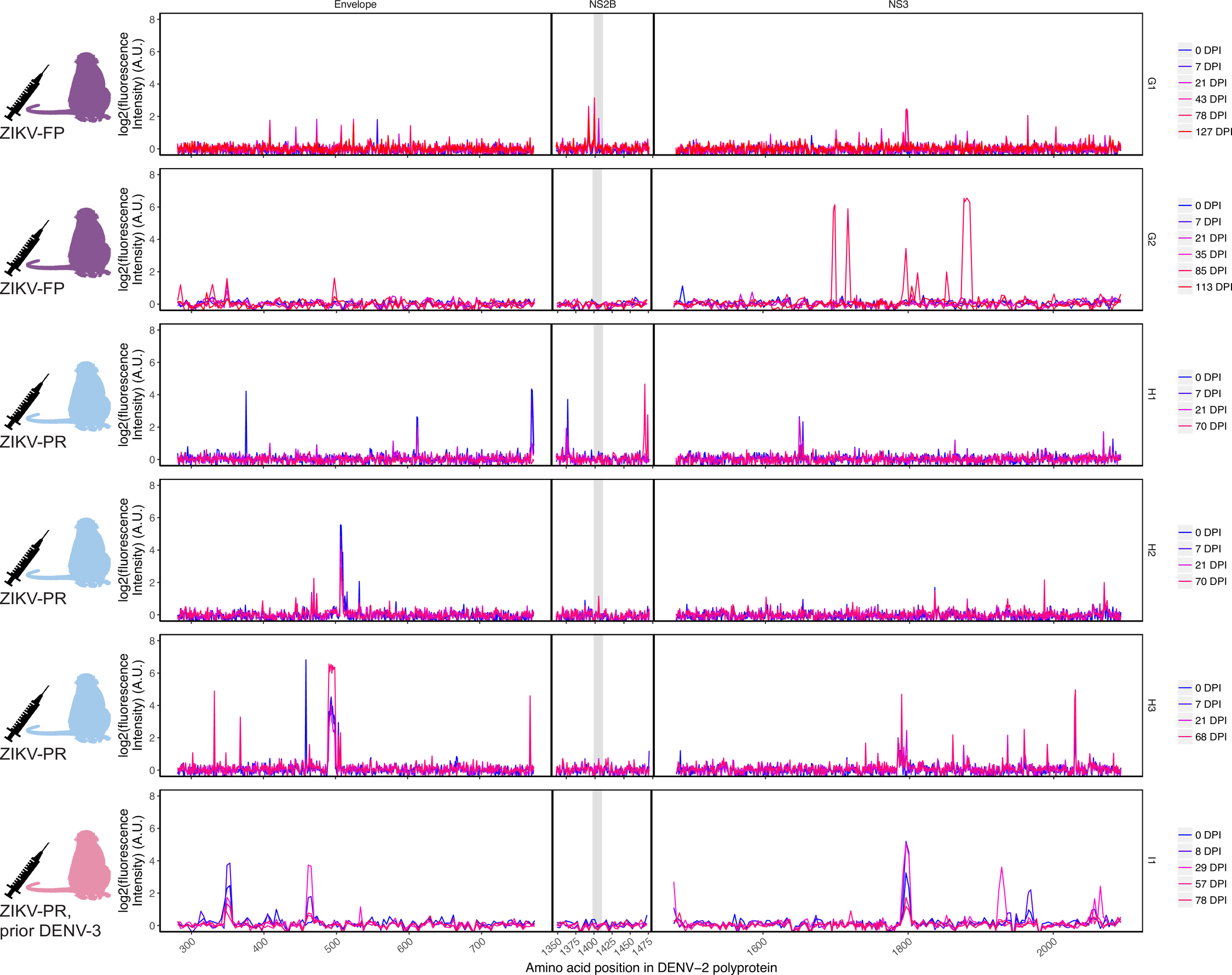

Supplement: S6 Fig — The region of the DENV-2 polyprotein corresponding to the ZIKV NS2B1427-1451RD25 epitope is highlighted in grey. (TIF) [file pntd.0006903.s007.tif]

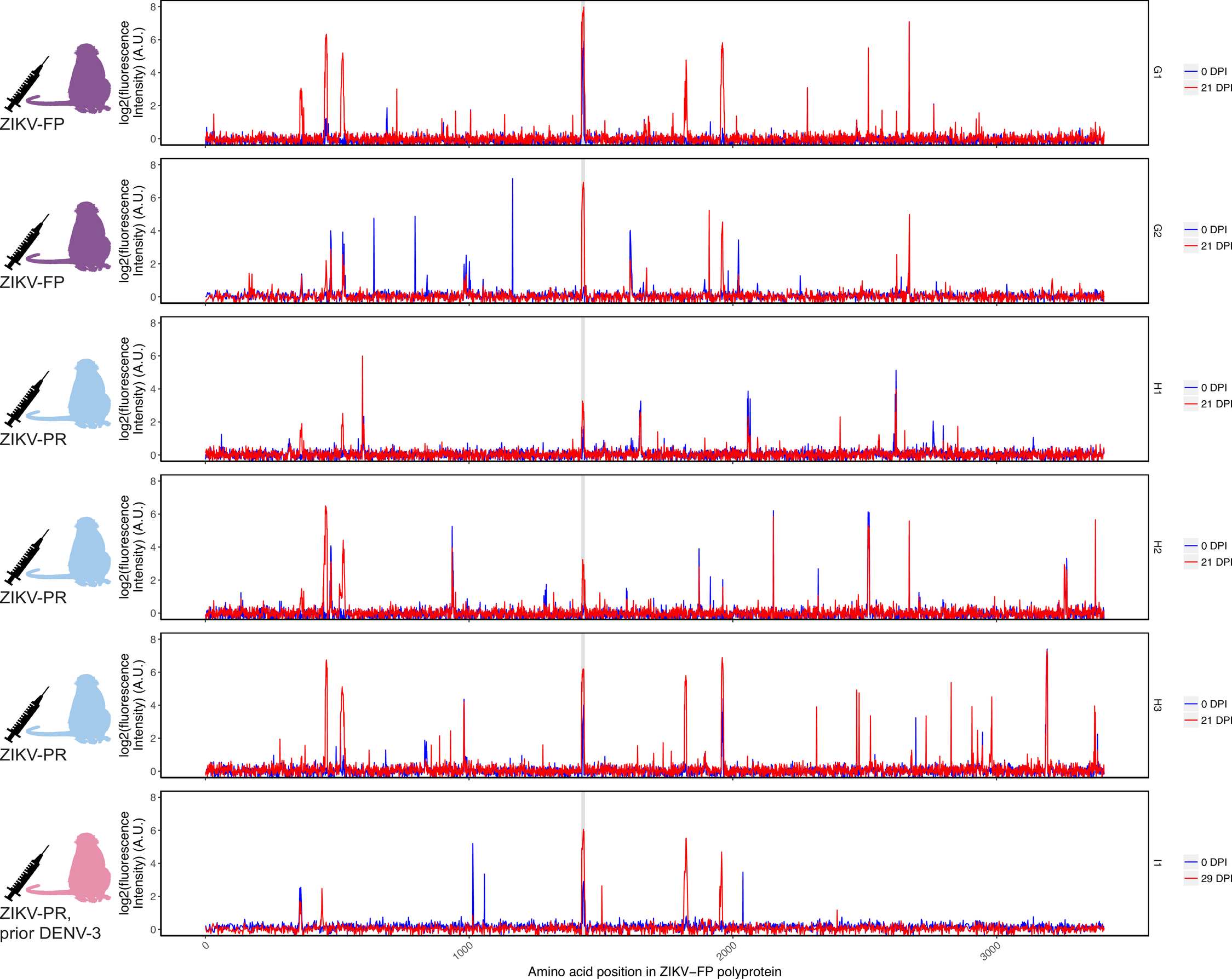

Supplement: S7 Fig — The NS2B1427-1451RD25 epitope is highlighted in grey. (TIF) [file pntd.0006903.s008.tif]

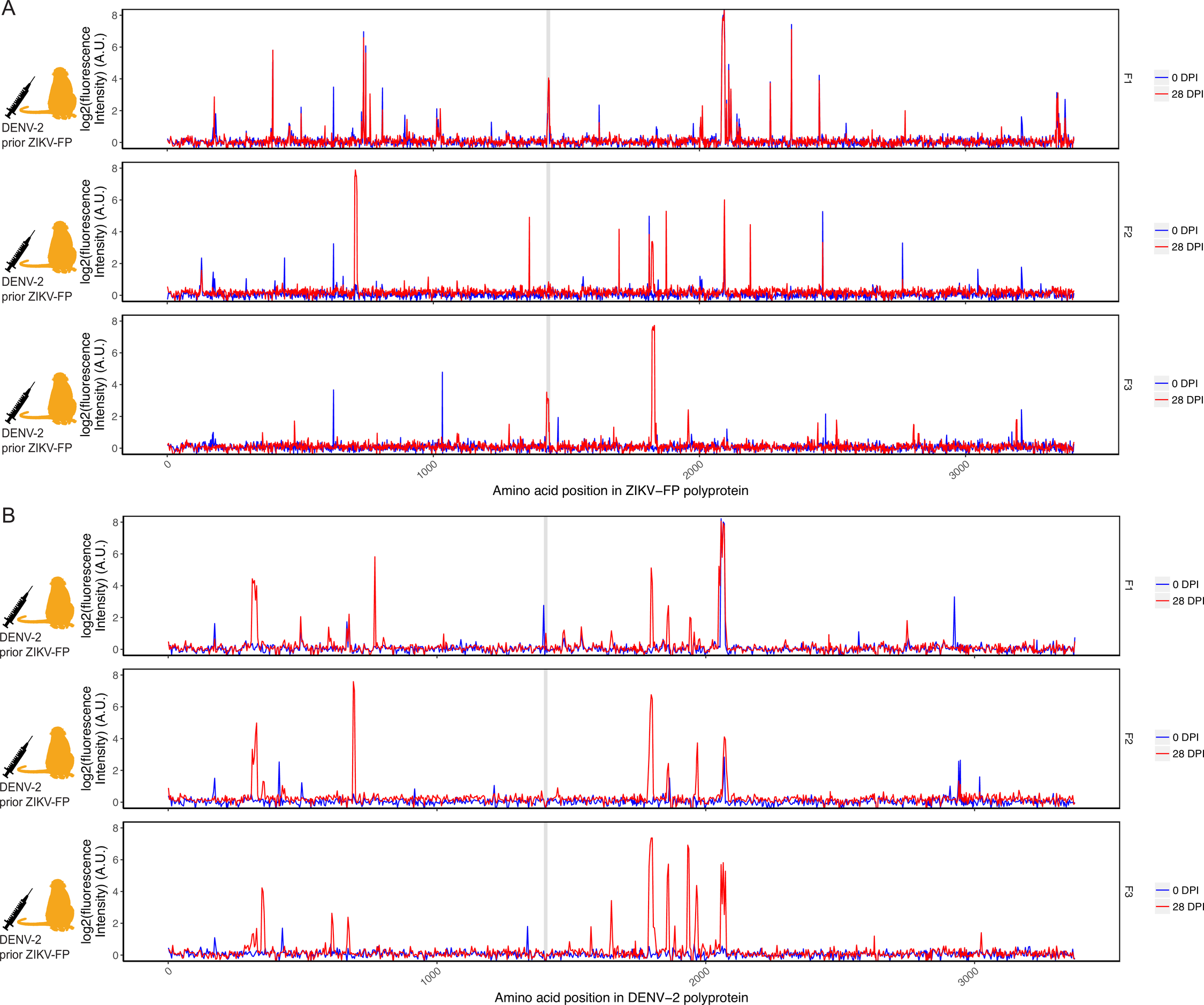

Supplement: S8 Fig — The NS2B1427-1451RD25 epitope in ZIKV-FP, and the corresponding area in DENV-2, is highlighted in grey. (TIF) [file pntd.0006903.s009.tif]
